# COMMO: a web server for the identification and analysis of consensus gene modules across multiple methods

**DOI:** 10.1093/bioinformatics/btad708

**Published:** 2023-11-23

**Authors:** Xiaojing Wu, Mingfei Han, Xinyu Song, Song He, Xiaochen Bo, Yunping Zhu

**Affiliations:** Basic Medical School, Anhui Medical University, Hefei 230022, China; National Center for Protein Sciences (Beijing), Beijing Proteome Research Center, Beijing Institute of Lifeomics, Beijing 102206, China; National Center for Protein Sciences (Beijing), Beijing Proteome Research Center, Beijing Institute of Lifeomics, Beijing 102206, China; Center for Artificial Intelligence in Medicine, Medical Innovation Research Division of Chinese, PLA General Hospital, Beijing 100853, China; Department of Bioinformatics, Institute of Health Service and Transfusion Medicine, Beijing 100850, China; Department of Bioinformatics, Institute of Health Service and Transfusion Medicine, Beijing 100850, China; Basic Medical School, Anhui Medical University, Hefei 230022, China; National Center for Protein Sciences (Beijing), Beijing Proteome Research Center, Beijing Institute of Lifeomics, Beijing 102206, China

## Abstract

**Summary:**

A variety of computational methods have been developed to identify functionally related gene modules from genome-wide gene expression profiles. Integrating the results of these methods to identify consensus modules is a promising approach to produce more accurate and robust results. In this application note, we introduce COMMO, the first web server to identify and analyze consensus gene functionally related gene modules from different module detection methods. First, COMMO implements eight state-of-the-art module detection methods and two consensus clustering algorithms. Second, COMMO provides users with mRNA and protein expression data for 33 cancer types from three public databases. Users can also upload their own data for module detection. Third, users can perform functional enrichment and two types of survival analyses on the observed gene modules. Finally, COMMO provides interactive, customizable visualizations and exportable results. With its extensive analysis and interactive capabilities, COMMO offers a user-friendly solution for conducting module-based precision medicine research.

**Availability and implementation:**

COMMO web is available at https://commo.ncpsb.org.cn/, with the source code available on GitHub: https://github.com/Song-xinyu/COMMO/tree/master.

## 1 Introduction

Functionally related gene modules detection is a critical step in the analysis of genome-wide gene expression profiles generated by high-throughput technologies. These modules can reveal functional relationships between genes and are useful for understanding the complexity of biological systems (Jojic *et al.* 2013). To date, numerous module detection methods have been developed, including clustering (Kluger *et al.* 2003), biclustering (Bergmann *et al.* 2003), matrix decomposition (Wang *et al.* 2021), and network inference methods (Huynh-Thu *et al.* 2010). However, many of these existing methods suffer from high parameter dependence and poor robustness (Saelens *et al.* 2018).

To address this issue, several studies (Brière *et al.* 2021, Qin *et al.* 2023) strongly advocated for the integration of modules from various methods to create more accurate and robust results. However, there is a lack of consensus clustering tools or websites that can integrate gene modules identified by different methods. First, existing gene module detection websites, such as ExpressVis (Liu *et al.* 2022a) and Hiplot (Li *et al.* 2022), only focus on a single clustering method. Second, existing consensus clustering websites, such as ICM (He *et al.* 2016) and COMSUC (He *et al.* 2021), are designed for disease subtyping rather than gene module detection. In summary, there is an urgent need for new tools that use consensus clustering to improve the detection of gene functionally related gene modules.

Here, we present COMMO (COnsensus Molecular MOdules), an interactive web server for biologists to identify and analyze consensus gene functionally related gene modules from different module detection methods. COMMO integrates eight representative module detection methods and two consensus clustering algorithms. Additionally, COMMO provides module-based functional enrichment and survival analysis, which gives researchers practical ways to assess module-disease associations and identify potential biomarkers. COMMO also provides a user-friendly interface and comprehensive result visualization, making it easy for researchers to explore and download results. To evaluate COMMO on clinical cohorts, we applied it to the TCGA breast cancer dataset and identified many well-known pathways and genes associated with breast cancer, as detailed in the Supplementary Materials. In conclusion, we believe that COMMO will be a valuable and convenient platform for combining independently generated clusters into more robust and reliable clusters.

## 2 Implementation

**Figure 1.**
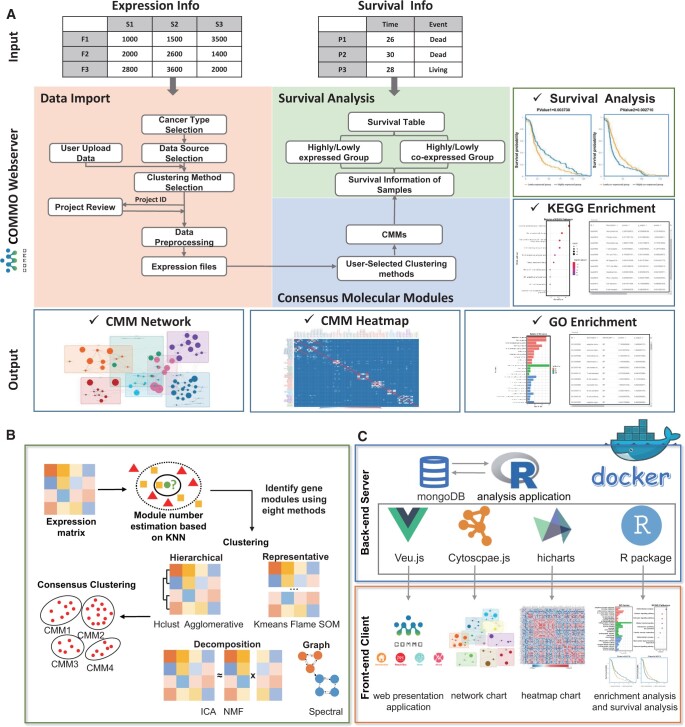
(A) The workflow of COMMO web server. (B) The process of COMMO identifying consensus molecular modules. (C) The network architecture of COMMO.

As shown in Fig. 1A, there are three steps to run COMMO in the web interface: (i) upload expression data to be analyzed, (ii) select module detection and consensus clustering methods, and (iii) explore interactive and exportable results. In the first step, users can upload their own private data or use mRNA and protein expression data across 33 cancer types from three public cancer data sources (TCGA, ICGC, and TARGET).

In the second step, COMMO identifies consensus molecular module (CMMs) in three steps (Fig. 1B). First, COMMO allows users to customize the number of clusters. If no cluster number is specified, COMMO will automatically determine the number of clusters using the k-nearest neighbor algorithm (see Supplementary Section S1.1). Second, eight clustering methods are used to identify gene modules, including FLAME (Fuzzy clustering by Local Approximation of Memberships) (Fu and Medico 2007), K-means (Timmerman *et al.* 2013), SOM (self-organizing mapping) (Wang *et al.* 2002), spectral clustering (Zhang *et al.* 2021), Agglomerative (Liu *et al.* 2022b), Hclust (Bu *et al.* 2022), NMF (non-negative matrix factorization) (Liefeld *et al.* 2023), and ICA (independent component analysis) (Hyvärinen 2013). Detailed descriptions of these eight methods can be found in the Supplementary Materials. We selected these methods because they demonstrated superior performance in a previous evaluation study (Saelens *et al.* 2018) and have reasonable runtimes. Finally, two consensus clustering methods, COCA (Cluster-Of-Cluster-Assignments) (The Cancer Genome Atlas Network 2012, Hoadley *et al.* 2014, Cabassi and Kirk 2020) and SuperCluster (Kandoth *et al.* 2013) are used to integrate different clustering results.

In the third step, COMMO provides five types of results, including CMM network maps, CMM heatmaps, module-based functional enrichment analysis, and two types of survival analysis (expression level-based survival analysis and module centrality-based survival analysis) to identify module genes that are associated with cancer patient survival (see Supplementary Methods for details). All graphics generated by the platform can be downloaded for demonstration or publishing, and all data in the table can be downloaded for later use. Notably, to save users time and ensure timely notification even in the event of network interruption, COMMO automatically emails users after a project is completed. Users can then download the result file within 30 days of receiving the email.

The architecture of COMMO is based on the client-server website design (Fig. 1C). The backend server focuses on computation, while the front-end client focuses on interactive visualization. COMMO uses a MongoDB database to store data, and the analysis application is written in R. The web presentation application is implemented in Vue.js. The backend server is deployed in Docker. Network maps are drawn with Cytoscape.js, heatmaps with Highcharts, GO and KEGG enrichment analyses with the ‘enrichGO’ and ‘enrichKEGG’ functions from the ‘clusterProfiler’ R package (Yu *et al.* 2012), and survival analysis with the ‘survfit’ function from the ‘survival’ R package. COMMO can be deployed locally or remotely on different platforms (including Windows and Linux). The source code is available on GitHub, along with deployment instructions.

## 3 Conclusion

In conclusion, COMMO is the first web server that can integrate multiple module detection methods to identify CMMs. It also offers extensive functional analysis and visualization capabilities for these modules and enables biologists to identify reliable consensus molecular modules and perform module-based precision medicine research. COMMO is available at https://commo.ncpsb.org.cn/.

## Supplementary Material

btad708_Supplementary_Data

## Data Availability

The COMMO server is designed to provide users with intuitive analysis of publicly available datasets of The Cancer Genome Atlas (TCGA), the International Cancer Genome Consortium (ICGC), and Therapeutically Applicable Research To Generate Effective Treatments (TARGET, https://ocg.cancer.gov/programs/target), and to allow users to upload their own data for analysis.
