# Supplementary material for: COMMO: a web server for the identification and analysis of consensus gene modules across multiple methods
Source: Bioinformatics. 2023 Nov 23;39(12):btad708. doi: 10.1093/bioinformatics/btad708 (PMC10713113; doi:10.1093/bioinformatics/btad708)
Supplement: btad708_Supplementary_Data [file btad708_supplementary_data.docx]

**COMMO: A web server for the identification and analysis of consensus gene modules based on multiple methods and datasets**

1. **Supplementary Methods**

**1.1 The number of clusters**

COMMO allows users to customize the number of clusters. If no cluster number is specified, COMMO will automatically determine the number of clusters using the k-nearest neighbor (KNN) algorithm. The KNN algorithm consists of three steps:

1. Find the k-nearest neighbors (k=10) for each gene and calculate their proximity based on the correlation coefficient.
2. Calculate the density for each gene using the proximity measurements. For instance, the density of gene $g_{0}$ is calculated as $d_{0}=\frac{1}{k}\sum_{i=1}^{k} p_{0,i}$, where $p_{0,i}$ represent the proximity between $g_{0}$ and its k-nearest neighbor $g_{e}$.
3. Define genes with the highest densities among their KNNs as potential clustering centers.

**1.2 Eight module detection methods**

**Fuzzy clustering by Local Approximation of Memberships (FLAME)**

FLAME cluster algorithm is a density-based clustering method. The main idea is to determine the quantity and shape of the cluster according to the distance and density between the samples. In this algorithm, first use all sample points as a cluster, and calculate the distance from other sample points for each sample point. Then, the threshold parameters and minimum samples are set to delete lower -density sample points to form different clusters. At the same time, for those isolation points that cannot be attributed to any clusters, they can be regarded as noise points or clusters alone. The advantage of FLAME cluster algorithm compared to other cluster algorithms is that it can adapt to the quantity and shape of the cluster adapting, and it has a good robustness for abnormal points.

**K-means Clustering (K-means)**

K-means clustering is one of the top 10 algorithms in data mining and one of the most popular clustering algorithms. K-means clustering aims to partition n observations into k clusters in which each observation belongs to the cluster with the nearest mean, serving as a prototype of the cluster.

**Self-Organizing Map Clustering (SOM)**

A SOM is a type of artificial neural network (ANN) that is trained using unsupervised learning to produce a low-dimensional (typically two-dimensional), discretized representation of the input space of the training samples, called a map, and is therefore a method to do dimensionality reduction. SOMs operate in two modes: training and mapping. “Training” builds the map using input examples (a competitive process, also called vector quantization), while “mapping” automatically classifies a new input vector.

**Spectral Clustering (Spectralclust)**

The Spectral Clustering is a clustering algorithm based on graph -based, which regards the data point as the vertex of the high -dimensional space and uses the similarity of the similarity between them. This algorithm reduces the dimension by calculating the feature vector, and then the classification of these vectors to obtain the final result. The Special Clustering has good clustering performance and explanatory, especially when dealing with non-spherical clustering problems.

**Agglomerative Clustering (Agglomerative)**

The Agglomerative clustering algorithm is a layered cluster algorithm from the bottom upwards. It considers each data point as a separate initial cluster, and then iterates the two nearest clusters to meet a certain stop criterion.

**Hierarchical Clustering (Hclust)**

Hclust is a method of cluster analysis which seeks to build a hierarchy of cluster. Strategies for Hclust generally fall into two types: 1) “bottom-up” approach: each observation starts in its own cluster, and pairs of clusters are merged as one moves up the hierarchy; 2) “top-down” approach: all observations start in one cluster, and splits are performed recursively as one moves down the hierarchy. In general, the merges and splits are determined in a greedy manner. The results of hierarchical clustering are usually presented in a dendrogram

**Independent Component Analysis (ICA)**

ICA cluster algorithm is a clustering method based on independent principles to find independent components in mixed signals. It assumes that the source signal is independent and non-Gaussian distribution, and determines the mixed matrix and independent components by iterative optimization and solution. In the problem of clustering, ICA can convert data to independent components, and then process these ingredients. Compared with the traditional clustering method, the ICA cluster does not require the number of clusters in advance, because it can automatically learn independent ingredients from the data. In addition, the ICA cluster also has good robustness and versatility, and is widely used in the fields of signal processing, image processing, and pattern recognition.

**Non-negative Matrix Factorization (NMF)**

NMF is a group of algorithms in multivariate analysis and linear algebra where a matrix V is factorized into (usually) two matrices W and H, with the property that all three matrices have no negative elements. This non-negativity makes the resulting matrices easier to inspect.

**1.3 Two consensus clustering algorithms**

Consensus clustering algorithms aim to combine the independently generated clusters into a more robust and reliable clustering. COMMO uses two algorithms for the consensus cluster: Cluster-Of-Cluster-Assignments (COCA) (Cabassi and Kirk, 2020; Hoadley, et al., 2014; Network, 2012; Wilkerson and Hayes, 2010) and Supercluster (Cancer Genome Atlas Research, et al., 2013) to incorporate the results of the different methods of clustering.

In the COCA analysis, we encoded the gene assignments of different clustering methods as a series of indicator variables, creating a matrix of 1s and 0s. We then used this matrix in the ConsensusClusterPlus R package (Wilkerson and Hayes, 2010) to identify patterns of relationships among the genes. ConsensusClusterPlus was run with 80% sample resampling and 1000 iterations of hierarchical clustering based on a Pearson correlation distance metric. For the SuperCluster analysis, we implemented the SuperCluster algorithm proposed by Kandoth et al (Cancer Genome Atlas Research, et al., 2013) using R code, which derives overall clusters by performing hierarchical clustering on the genes based on their cluster assignments in different clustering methods.

**1.4 Analysis results**

Users can import their own data or load public data sets, and select multiple clustering algorithms and a consensus clustering algorithm to identify consensus molecular modules (CMMs). COMMO provides five types of results, each of which is displayed in interactive graphics and tables. All graphics generated by the platform can be downloaded for demonstration or publishing, and all data in the table can be downloaded for later use. The details of these results are as follows:

**(1) CMM network**

The boxes in the CMM Network (Supplementary Fig 2A) represent consensus molecular modules, and the nodes represent clusters generated by individual clustering methods. The size of each node corresponds to the number of genes involved in the cluster. The width of each edge represents the Jaccard similarity coefficient between different clusters, and the transparency of each edge represents the negative log10 p-value from the hypergeometric test. Let $S$ denote the total number of genes, $M$ denote the number of genes in cluster A, $N$ denote the number of genes in cluster B, and $c$ denote the number of genes in the overlap between clusters A and B. The hypergeometric test p-value of clusters A and B is calculated as:

$$p=\sum_{i=c}^{min(M,N)} \frac{\binom{N}{i}\binom{S-N}{M-i}}{\binom{S}{M}}$$

The p-values are adjusted for multiple testing by Benjamini–Hochberg (BH) correction (Benjamini and Hochberg, 1995).

The CMM network can be freely zoomed in and out using the mouse wheel. Additionally, both clusters generated by individual clustering methods (circles) and consensus gene modules (boxes) can be dragged and adjusted to prevent overlap before exporting the image. This makes it easy to see all the features in the maps, even those that are small or hidden. Clicking on a node will display a list of genes in that node in the right sidebar, clicking on a CMM box will display a list of genes in that CMM in the right sidebar, and clicking on a gene of interest in the list will link to NCBI.

**(2) CMM heatmap**

The horizontal and vertical axes of the CMM heatmap represent all clusters generated by different clustering methods. Axis labels are color-coded by CMM, such that axis labels associated with the same CMM are displayed in the same color, while axis labels associated with different CMMs are displayed in different colors. The color of each block represents the Jaccard similarity coefficient between the corresponding clusters. We observed that clusters associated with the same CMM tend to have higher Jaccard similarity coefficients and form numerous red and white blocks (yellow boxes in Supplementary Fig 2B). Users can change the heatmap color in the Control Panel Options tab. Clicking a color block displays the genetic message of the corresponding data block in the Control Panel Gene Information tab on the right.

**(3) Functional enrichment analysis**

Functional enrichment analysis is performed based on KEGG and Gene Ontology (GO) databases. Significant pathways can help researchers understand the relationship between genes and metabolic pathways, and how they affect different biological processes and diseases. GO enrichment analysis can help researchers understand gene function and reveal the interactions between different genes in CMM. The GO and KEGG enrichment analyses are performed using the ‘enrichGO’ and ‘enrichKEGG’ functions from the ‘clusterProfiler’ R package (Yu, et al., 2012). Both functions use the hypergeometric test to identify significant GO terms and pathways with default parameters, including a p-value cutoff of 0.05, a q-value cutoff of 0.2, a minimum of 10 genes per GO term or pathway, and a maximum of 500 genes.

**(4) Survival analysis**

COMMO provides two survival analysis approaches, expression level-based survival analysis and expression centrality-based survival analysis, to identify genes that are associated with the survival of cancer patients. The expression level-based survival analysis, which has been widely utilized in web-based survival analysis tools (Borcherding, et al., 2018; Goswami and Nakshatri, 2013; Gyorffy, et al., 2010; Xie, et al., 2019), aims to assess the correlation between gene expression and patient survival. This method ranks patients according to the expression of a particular gene. The log-rank test is then used to compare the survival of patients in the top and bottom halves (or quartiles) of the ranking (groups 1 and 2 in Supplementary Fig 1A). If a significant difference is observed, the gene is significantly associated with patient prognosis and survival.


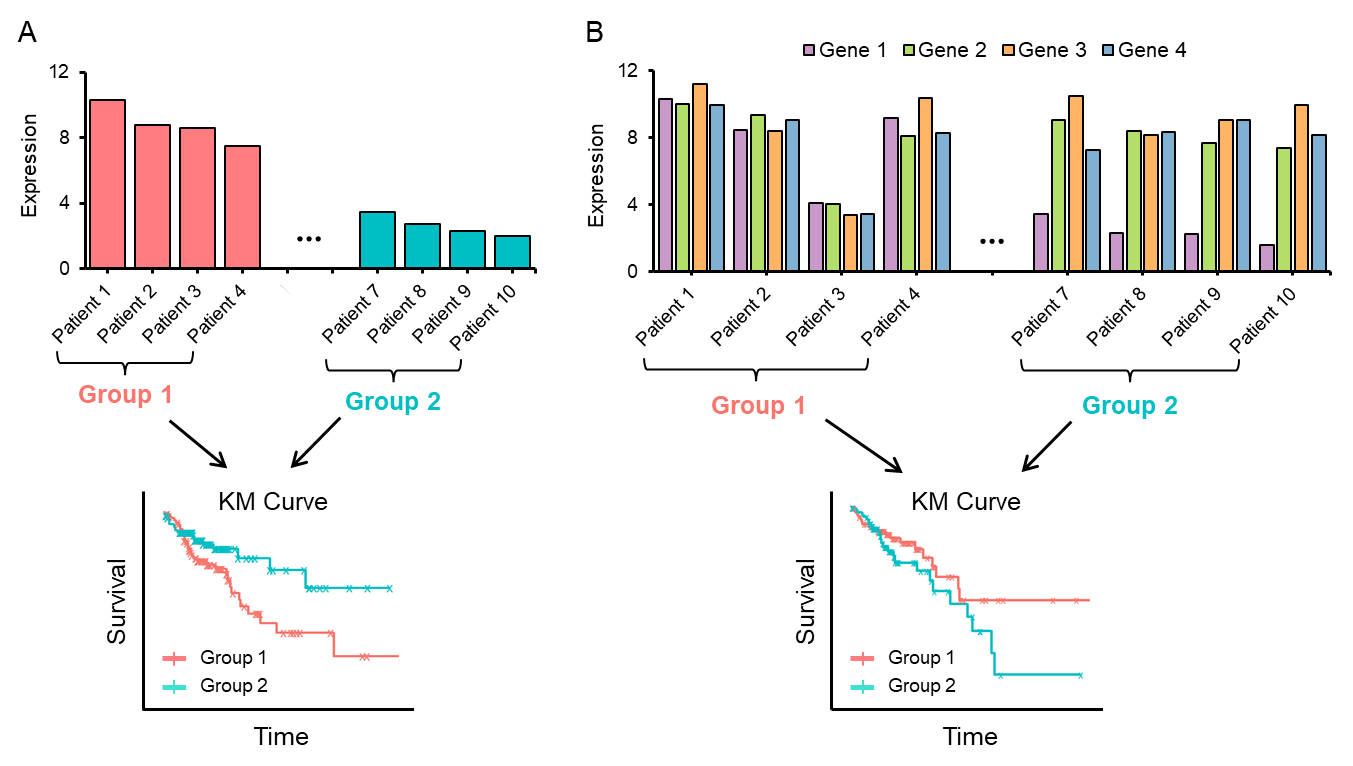


**Supplementary Figure 1.** Schematic of (A) expression level-based survival analysis and (B) expression centrality-based survival analysis.

The expression centrality-based survival analysis aims to explore the association between gene expression similarity and patient survival. Given a gene module of $M=(G,P)$, where $G=\left\{ g_{1},\ldots,g_{m} \right\}$ represents the genes included in $M$ and $P=\left\{ p_{1},\ldots,p_{n} \right\}$ represents all patient samples. Each gene expression vector is denoted as $V_{i}=\left\{ v_{i,1},\ldots,v_{i,n} \right\}$, where $i\in[1,m]$. First, z-score normalization is performed on each gene expression vector to ensure that different genes can be compared. Second, the expression centrality of each gene is quantified as the sum of the normalized expression differences between the gene and all other genes in the module. For instance, the expression centrality of $g_{1}$ in patient $j$ ($j\in[1,n]$) equals $\sum_{i=2}^{m} {(z_{1,j}-z_{i,j})}^{2}$, where $z$ represents the z-score normalized expression value.

Patients are then ranked based on the expression centrality of $g_{1}$. As shown in Supplementary Fig 1B, $g_{1}$ has a similar expression pattern to other genes in the top-ranked patients (group 1), but a different expression pattern to other genes in the bottom-ranked patients (group 2). This suggests that $g_{1}$ may be dysfunctional in group 2, as expression similarity is a well-established indicator of functional association. A significant survival difference between groups 1 and 2 indicates that this dysfunction could have a significant impact on cancer progression.

Previous research (Chen, et al., 2023) has shown that both the expression level of individual genes and the expression similarity of multiple genes have a significant impact on patient survival. Hence, we strongly advocate for the combination of expression level-based and expression centrality-based survival analyses as a promising avenue in future cancer research.

**1.5 Method evaluation**

Assume the data have been clustered via any technique into $k$ clusters. For gene $i\in C_{I}$ (gene $i$ in the cluster $C_{I}$), let

$$a\left( i \right)=\frac{1}{\left| C_{I} \right|-1}\sum_{j\in C_{I},i\neq j} r(i,j)$$

be the mean similarity between $i$ and all other genes in the same cluster, where $\left| C_{I} \right|$ is the number of points belonging to cluster $C_{I}$, and $r(i,j)$ is the Pearson correlation coefficient between genes $i$ and $j$ in the cluster $C_{I}$. We can interpret $a\left( i \right)$ as a measure of how well $i$ is assigned to its cluster. We then define the mean similarity of gene $i$ to cluster $C_{J}$ as the mean of the Pearson correlation coefficients between $i$ and all genes in $C_{J}$ (where ${C_{I}\neq C}_{J}$). For each gene $i\in C_{I}$, we define

$$b\left( i \right)=\max_{J\neq I} \frac{1}{\left| C_{J} \right|}\sum_{j\in C_{J}} r(i,j)$$

to be the largest mean similarity between $i$ to all genes in any other cluster. The cluster with this largest mean similarity is said to be the "neighboring cluster" of $i$ because it is the next best fit cluster for gene $i$. We now define a silhouette score for one gene $i$:

$$s\left( i \right)=\frac{a\left( i \right)-b\left( i \right)}{max\left\{ a\left( i \right),b\left( i \right) \right\}}$$

where $\left| C_{I} \right|>1$, and $s\left( i \right)=0$ if $\left| C_{I} \right|=1$. An $s\left( i \right)$ close to 1 means that the data is appropriately clustered. If $s\left( i \right)$ is close to -1, then by the same logic we see that $i$ would be more appropriate if it was clustered in its neighboring cluster. The mean $s\left( i \right)$ of all genes ($s_{mean}$) in the expression data is a measure of how appropriately the data have been clustered. To ensure comparability between methods, we normalize the score ($s_{mean}$) by subtracting the average score of 500 random permutations of the module genes.

1. **Supplementary Results**

**2.1 A case study of breast cancer datasets**

To evaluate the performance of COMMO on real-world data, we selected eight clustering methods (Hclust, K-means, NMF, SOM, SpectralClust, FLAME, Agglomerative, and ICA) and the COCA consensus clustering method to re-analyze a published breast cancer dataset containing transcription expression data of 20,530 genes in 508 samples, along with the corresponding follow-up overall survival information. COMMO integrated the modules identified by different methods into seven CMMs (Supplementary Fig 2A-B).

**Supplementary Figure 2.** (A) CMM network for breast cancer. Nodes: gene clusters generated by individual clustering methods. Edges: Jaccard similarity coefficients between clusters. Boxes: consensus gene modules. (B) CMM heatmap for breast cancer. Horizontal and vertical coordinates: gene clusters generated by individual clustering methods. Color of each block: Jaccard similarity coefficient between clusters.

We first compared the performance of eight clustering methods and two consensus clustering methods on TCGA breast cancer data using the silhouette score (see Supplementary Methods section 1.5). We found that the two consensus clustering methods achieved higher silhouette scores than the eight module detection methods (Supplementary Fig 3), indicating that the integration of independent clusters significantly improved the within-module similarity and between-module dissimilarity.


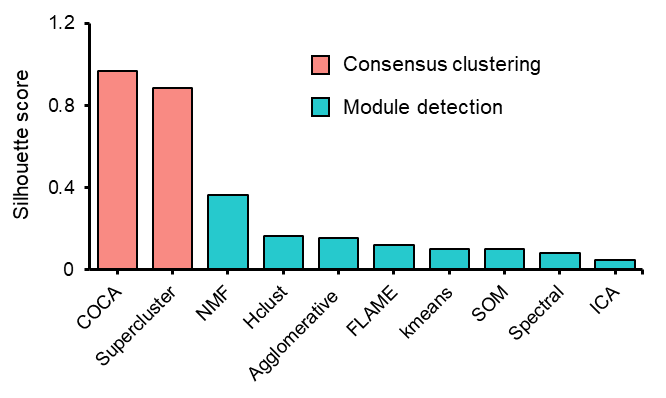


**Supplementary Figure 3.** The silhouette scores of eight clustering algorithms and two consensus clustering methods.

We selected CMM 1 (216 genes) and CMM4 (122 genes) for in-depth study. Functional enrichment analysis of CMM1 (Supplementary Fig 4A-B) revealed enrichment in endocrine system pathways, including estrogen signaling, oxytocin signaling, and cortisol synthesis and secretion. Literature review showed that these pathways play critical and complex roles in breast cancer. For example, estrogen signaling modulates the expression and activity of many different targets in breast cancer, ultimately promoting or inhibiting cancer progression (Lipovka and Konhilas, 2016). Emerging evidence suggests that oxytocin signaling also plays a role in breast cancer development and progression (Liu, et al., 2020). Cortisol may initiate protumorigenic changes during periods of stress by affecting estrogen activity (Antonova, et al., 2011). Additionally, we identified two genes, PGR and EGOT, which were significant in both expression level-based and expression centrality-based survival analyses (Supplementary Fig 4C-D). PGR is a gene that encodes the progesterone receptor protein, a steroid receptor that plays a role in the pathogenesis of breast cancer (Kim, et al., 2013). LncRNA EGOT decreases breast cancer cell viability and migration via inactivation of the Hedgehog pathway (Qiu, et al., 2020).


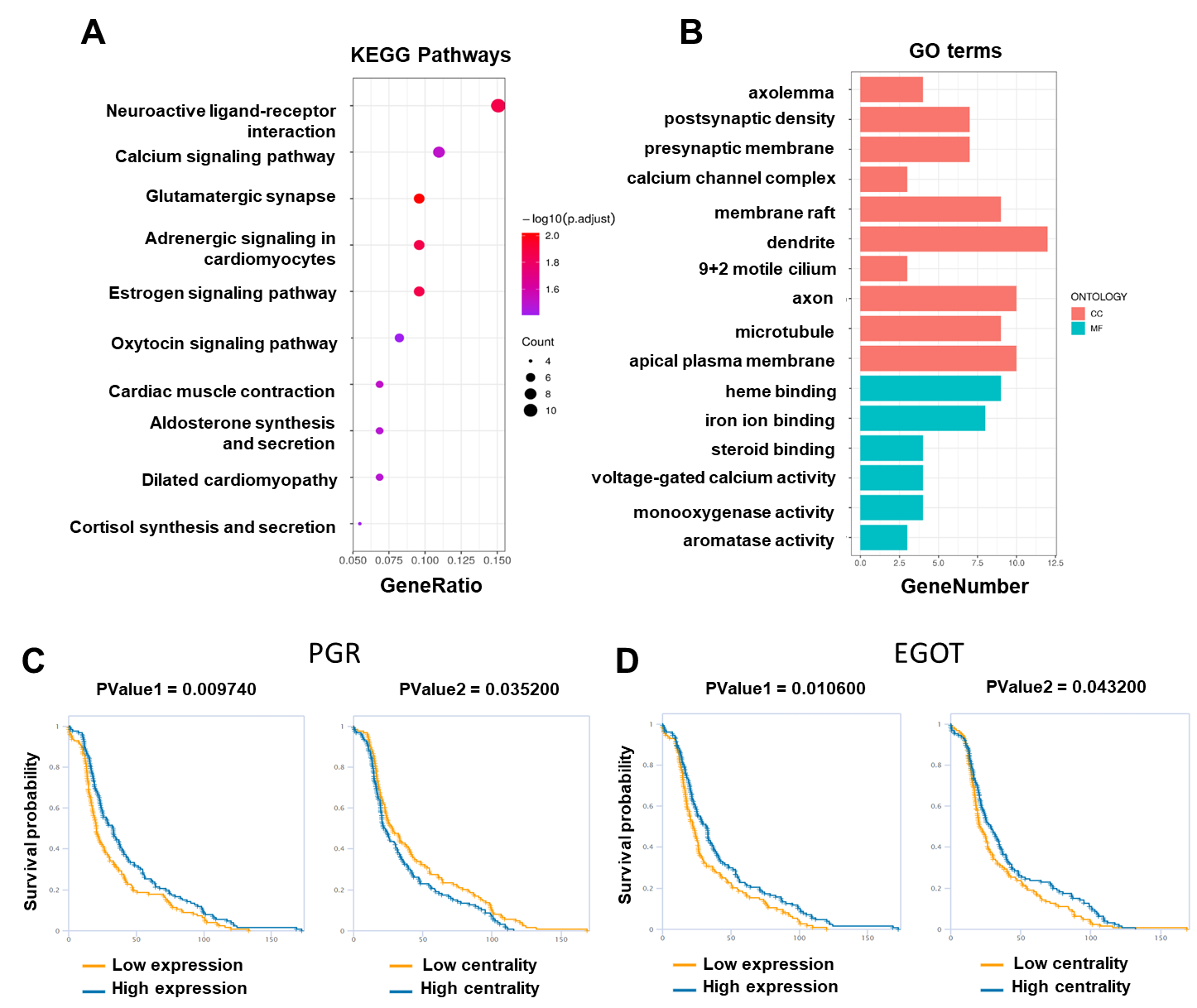


**Supplementary Figure 4.** KEGG pathways (A) and GO terms (B) identified by enrichment analysis of CMM1. (C) Survival gene PGR identified by expression level-based survival analysis and expression centrality-based survival analysis (D) Survival gene EGOT identified by expression level-based survival analysis and expression centrality-based survival analysis.

Functional enrichment analysis of CMM4 identified numerous immune-related pathways, including NF-kappa B signaling, T cell receptor signaling, B cell receptor signaling, immune response, inflammatory response, and chemokine activity (Supplementary Fig 5A-B). Breast tumor progression is known to be either prevented by antitumor immunity or exacerbated by proinflammatory cytokines released mainly by immune cells (Amens, et al., 2021). The expression level-based and expression centrality-based survival analyses identified two known prognostic biomarkers in breast cancer: GZMK and PRKCQ. Literature exploration revealed that GZMK is differentially expressed in T cells isolated from breast tumors compared to peripheral blood T cells (Plitas, et al., 2016), and PRKCQ encodes a serine/threonine kinase that promotes breast cancer growth, anoikis resistance, epithelial-mesenchymal transition (EMT), and invasion (Byerly, et al., 2020). In summary, the analysis of CMM1 and CMM4 demonstrates COMMO's great potential for identifying functionally related gene modules and advancing our understanding of cancer mechanisms.


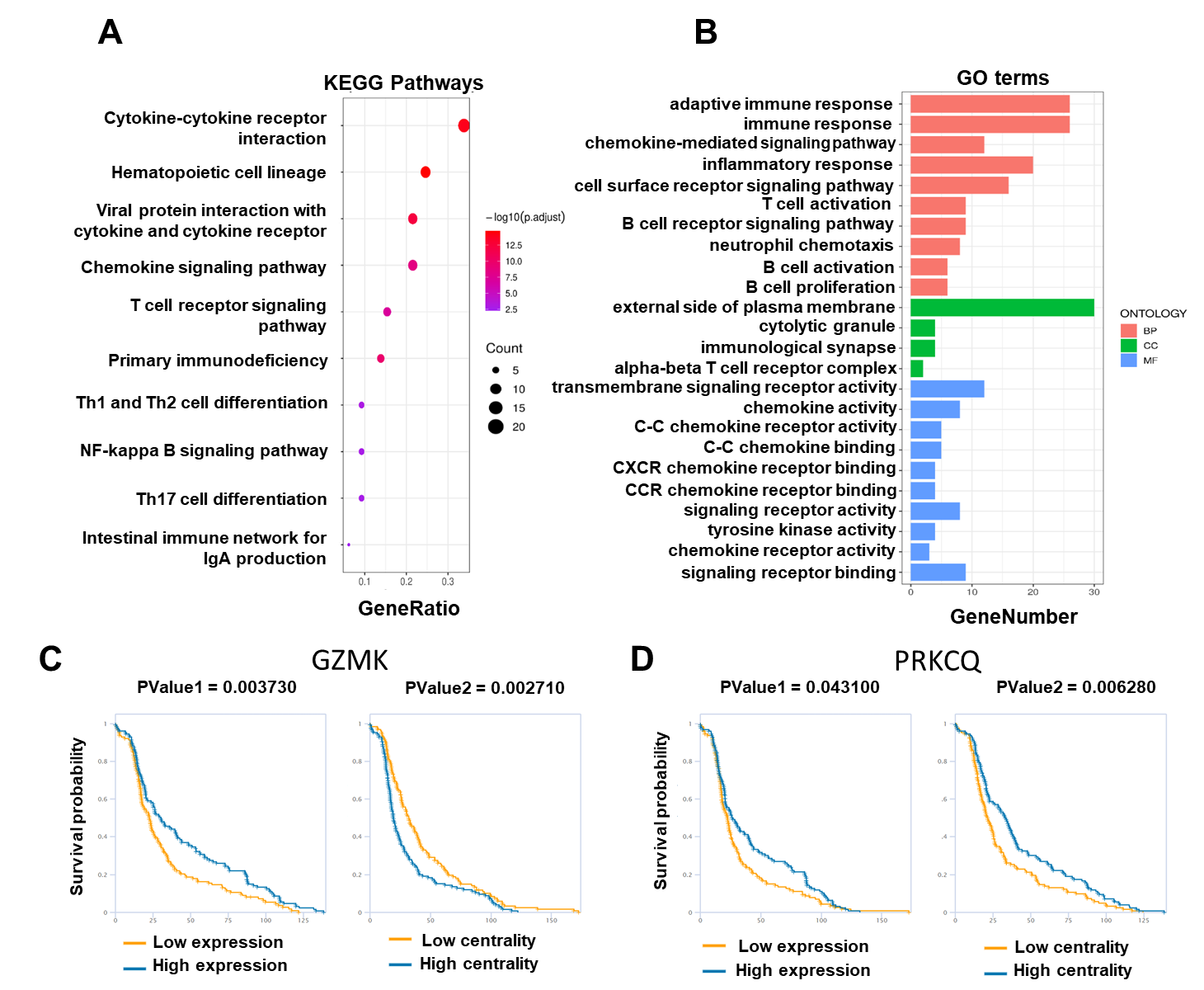


**Supplementary Figure 5.** KEGG pathways (A) and GO terms (B) identified by enrichment analysis of CMM4. (C) Survival gene GZMK identified by expression level-based survival analysis and expression centrality-based survival analysis (D) Survival gene PRKCQ identified by expression level-based survival analysis and expression centrality-based survival analysis.

Notably, expression level-based survival analysis of CMM1 only identified ten genes significantly correlated with breast cancer survival, while expression centrality-based survival analysis identified 35 survival genes. This indicated that in many cases, patient prognosis is not decided by the expression of a single gene but by the synergistic effects of multiple co-regulated genes. We strongly advocate for incorporating expression centrality into survival analysis as an essential complement to individual gene expression.

**Reference**

Amens, J.N., Bahcecioglu, G. and Zorlutuna, P. Immune System Effects on Breast Cancer. *Cell Mol Bioeng* 2021;14(4):279-292.

Antonova, L., Aronson, K. and Mueller, C.R. Stress and breast cancer: from epidemiology to molecular biology. *Breast Cancer Res* 2011;13(2):208.

Benjamini, Y. and Hochberg, Y. Controlling the False Discovery Rate: A Practical and Powerful Approach to Multiple Testing. *Journal of the Royal Statistical Society: Series B (Methodological)* 1995;57(1):289-300.

Borcherding, N.*, et al.* TRGAted: A web tool for survival analysis using protein data in the Cancer Genome Atlas. *F1000Res* 2018;7:1235.

Byerly, J.H., Port, E.R. and Irie, H.Y. PRKCQ inhibition enhances chemosensitivity of triple-negative breast cancer by regulating Bim. *Breast Cancer Res* 2020;22(1):72.

Cabassi, A. and Kirk, P.D.W. Multiple kernel learning for integrative consensus clustering of omic datasets. *Bioinformatics (Oxford, England)* 2020;36(18):4789-4796.

Cancer Genome Atlas Research, N.*, et al.* Integrated genomic characterization of endometrial carcinoma. *Nature* 2013;497(7447):67-73.

Chen, X.*, et al.* Identification of functional gene modules by integrating multi-omics data and known molecular interactions. *Frontiers in genetics* 2023;14:1082032.

Goswami, C.P. and Nakshatri, H. PROGgene: gene expression based survival analysis web application for multiple cancers. *J Clin Bioinforma* 2013;3(1):22.

Gyorffy, B.*, et al.* An online survival analysis tool to rapidly assess the effect of 22,277 genes on breast cancer prognosis using microarray data of 1,809 patients. *Breast Cancer Res Treat* 2010;123(3):725-731.

Hoadley, K.A.*, et al.* Multiplatform analysis of 12 cancer types reveals molecular classification within and across tissues of origin. *Cell* 2014;158(4):929-944.

Kim, J.J., Kurita, T. and Bulun, S.E. Progesterone action in endometrial cancer, endometriosis, uterine fibroids, and breast cancer. *Endocr Rev* 2013;34(1):130-162.

Lipovka, Y. and Konhilas, J.P. The complex nature of oestrogen signalling in breast cancer: enemy or ally? *Biosci Rep* 2016;36(3).

Liu, H.*, et al.* The oxytocin receptor signalling system and breast cancer: a critical review. *Oncogene* 2020;39(37):5917-5932.

Plitas, G.*, et al.* Regulatory T Cells Exhibit Distinct Features in Human Breast Cancer. *Immunity* 2016;45(5):1122-1134.

Qiu, S.*, et al.* LncRNA EGOT decreases breast cancer cell viability and migration via inactivation of the Hedgehog pathway. *FEBS Open Bio* 2020;10(5):817-826.

The Cancer Genome Atlas Network. Comprehensive molecular portraits of human breast tumours. *Nature* 2012;490(7418):61-70.

Wilkerson, M.D. and Hayes, D.N. ConsensusClusterPlus: a class discovery tool with confidence assessments and item tracking. *Bioinformatics* 2010;26(12):1572-1573.

Xie, L.*, et al.* OSacc: Gene Expression-Based Survival Analysis Web Tool For Adrenocortical Carcinoma. *Cancer Manag Res* 2019;11:9145-9152.

Yu, G.*, et al.* clusterProfiler: an R package for comparing biological themes among gene clusters. *Omics* 2012;16(5):284-287.
